# Automatic delineation of glacier grounding lines in differential interferometric synthetic-aperture radar data using deep learning

**DOI:** 10.1038/s41598-021-84309-3

**Published:** 2021-03-02

**Authors:** Yara Mohajerani, Seongsu Jeong, Bernd Scheuchl, Isabella Velicogna, Eric Rignot, Pietro Milillo

**Affiliations:** 1 Department of Earth System Science, University of California Irvine, Irvine, CA 92697 USA; 2 eScience Institute and Department of Civil and Environmental Engineering, University of Washington, Seattle, WA 98195 USA; 3 Jet Propulsion Laboratory, Pasadena, CA 91109 USA

**Keywords:** Cryospheric science, Climate sciences

## Abstract

Delineating the grounding line of marine-terminating glaciers—where ice starts to become afloat in ocean waters—is crucial for measuring and understanding ice sheet mass balance, glacier dynamics, and their contributions to sea level rise. This task has been previously done using time-consuming, mostly-manual digitizations of differential interferometric synthetic-aperture radar interferograms by human experts. This approach is no longer viable with a fast-growing set of satellite observations and the need to establish time series over entire continents with quantified uncertainties. We present a fully-convolutional neural network with parallel atrous convolutional layers and asymmetric encoder/decoder components that automatically delineates grounding lines at a large scale, efficiently, and accompanied by uncertainty estimates. Our procedure detects grounding lines within 232 m in 100-m posting interferograms, which is comparable to the performance achieved by human experts. We also find value in the machine learning approach in situations that even challenge human experts. We use this approach to map the tidal-induced variability in grounding line position around Antarctica in 22,935 interferograms from year 2018. Along the Getz Ice Shelf, in West Antarctica, we demonstrate that grounding zones are one order magnitude (13.3 ± 3.9) wider than expected from hydrostatic equilibrium, which justifies the need to map grounding lines repeatedly and comprehensively to inform numerical models.

## Introduction

The grounding line is the transition boundary where land ice meets with ocean waters along the periphery of Greenland and Antarctica^1,2^. Upstream of the grounding line, ice rests on land and is resisted by bedrock and sediments and along valley walls. Downstream of the grounding line, ice is no longer grounded; it floats in the ocean waters and melts in contact with these waters. This boundary is fundamental to the study of ice sheets for many reasons. First, it is the boundary where we measure ice mass fluxes into the ocean^3^. Second, ice experiences a fundamental change in force balance at that boundary, transitioning from basal friction at the bed to frictionless motion on seawater at sea^4^, so it is essential to inform ice sheet numerical models about the location of the grounding line. At the grounding line, ice traverses hydrostatic equilibrium but bending stresses maintain ice below hydrostatic equilibrium over considerable distances seaward (10 km) until ice reaches full hydrostatic equilibrium in seawater^5^, which is important to consider when interpreting data from altimetry missions. Third, the surface slope is typically 1% at the grounding line, so a 1-m change in ice thickness translates into a 100-m horizontal migration of hydrostatic equilibrium^1^, therefore the grounding line position is a sensitive indicator of glacier thinning. Fourth, the grounding line is challenging to detect from above, it leaves little evidence in optical imagery^6^, it is elusive from the ground, and historically has been misplaced with errors ranging from kilometers to 100 km^1,5,7–9^.

Grounding lines are detected in several ways using remote sensing data^1^, but the most efficient and precise method is satellite interferometric synthetic-aperture radar (InSAR). InSAR documents the motion of ice in the line of sight of the radar illumination with millimeter precision. Provided enough crossing tracks of InSAR data, this technique maps ice motion in vector form^10^. By differencing two consecutive radar interferograms spanning the same interval, it measures the short term variability in ice motion associated to processes such as glacier subsidence in response to subglacial lake drainage or tidal motion in response to changes in ocean tide. Because differential InSAR (DInSAR) combines four epochs to “image” the grounding line, it mixes four different tidal displacements, but the data provide a snapshot of grounding line positions over considerable distances, i.e. hundreds of km, anywhere around the continent, and repeatedly over time.

**Figure 1:**
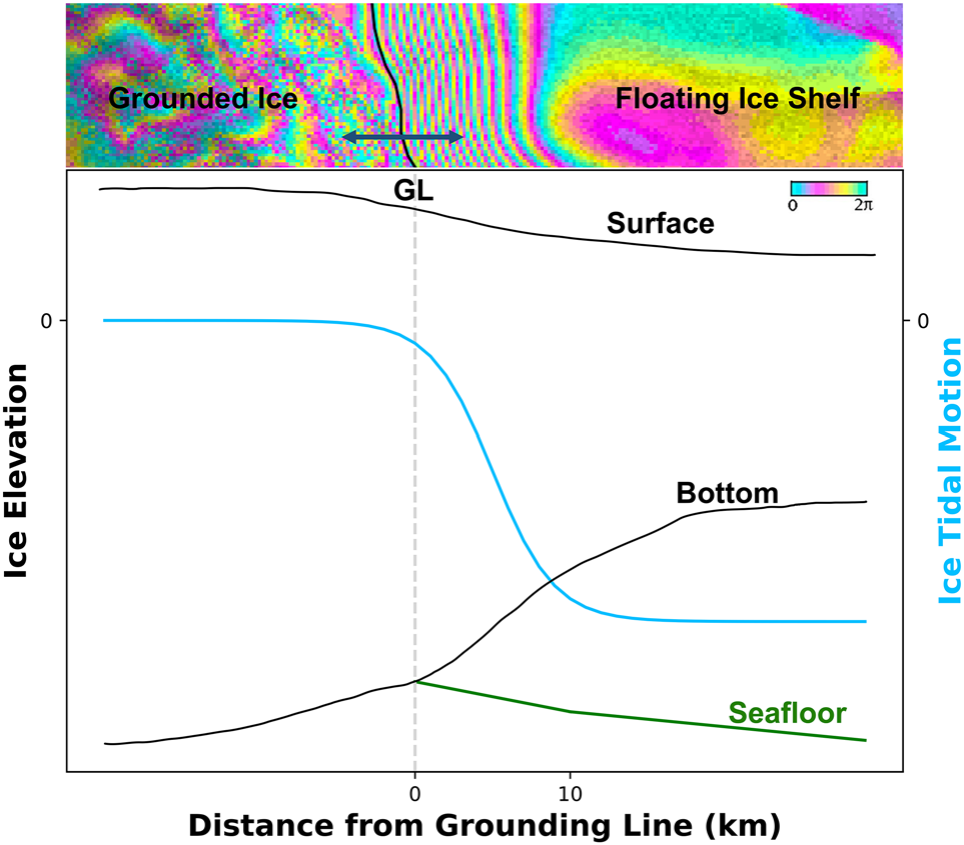
Schematic of the ice surface, ice bottom elevation, sea floor depth, and vertical tidal motion of the ice in the grounding zone versus a differential SAR interferogram on top. The grounding line (GL), represented by the vertical gray dashed line, is at the inner-most fringe of the grounded ice side. The differential motion in the tidal flexing zone produces a dense fringe pattern used by the neural network to identify grounding lines. The direction of horizontal migration of the GL is shown by the double-sided dark blue arrow.

At the grounding line, the DInSAR data reveals a step in vertical motion that is characteristic of the visco-elastic bending of ice afloat in ocean waters across the flexure zone (Fig. 1). The inner limit of the DInSAR signal, or inner interferometric fringe, marks the grounding line position. The outer limit marks the limit of the flexure zone where the glacier adapts to flotation. Here, we are most interested in the grounding line position.

To digitize the grounding lines, Rignot et al. (1998)^11^ fit the data with an elastic model, which was generalized in two dimensions^12^. The modeling identified the limit of tidal flexing, which is a proxy for the grounding line. While this approach worked well for smooth grounding line delineations, it failed in the presence of noise, required information on ice flow direction, and could not be automated or scaled to large areas. Instead, human experts have resorted to the practice of performing the work by hand, in part to resolve complex topological problems appearing in certain situations. For instance, interferometric fringes may be missing due to a loss of phase coherence, stretch out significantly along deep, narrow valleys, under-sampled, or not well resolved. While the manual approach has been acceptable for a few DInSAR pairs of Greenland and Antarctic glaciers, it has become cumbersome as more data become available from more SAR satellites. Furthermore, interpretation may vary among human experts and errors are not documented.

The European Space Agency launched the Sentinel-1a/b constellation in 2014 and 2016, respectively, as part of the European Union’s Copernicus Earth Observation program to provide InSAR data over the ice sheets systematically for the first time and for decades to come with a 6-day repeat cycle (for the constellation) or a 12-day repeat cycle (for a single satellite)^13,14^. This situation contrasts with the ERS-1/2 mission which provided only a handful of DInSAR data to analyze. Digitizing Sentinel-1a/b DInSAR data is impractical by hand on a large scale.

Here, we present a novel approach to the automated delineation of grounding line which uses a machine learning (ML) algorithm. After introducing the training and evaluation data sets, we discuss the ML architecture employed to capture the grounding line, the evaluation of the results and estimation of errors, and demonstrate its use at the large scale around Antarctica with thousands of interferograms.

## Results

### DInSAR data

We employ DInSAR observations from the Sentinel-1a/b satellite captured in year 2018. The radar operates at the C-band (radar wavelength is 5.6 cm) and each interferometric fringe or 360 degree variation in phase corresponds to a 28 mm motion of the icy surface along the line of sight of the radar. Each interferogram is 250 km by 300 km in size, geocoded at 100 m spacing. Each sector of the grounding line is covered by several repeat tracks. The geocoded DInSAR data are first manually interpreted to serve as training and evaluation datasets, as outlined below. In total, we analyze 252 DInSAR-delineation pairs on the Getz Ice Shelf for training. To train the neural network, we use both the phase and coherence of the signal. Furthermore, we analyze 19,510 DInSAR interferograms with 6-day repeat between the pairs acquired from 22 Sentinel -1a/b tracks, and 3425 DInSAR interferograms with 12-day repeat between the pairs acquired from 23 Sentinel-1a/b tracks, totalling 22,935 interferograms over Antarctica in 2018. The 12-day tracks were trimmed to exclude in-land tiles far from the grounding zone to reduce the processing time. However, by including all the tiles from the 6-day tracks we demonstrate that the network is capable of dealing with areas without grounding lines. The detailed break-down of the number of interferograms and tiles is listed in Table S1.

We rasterize the manually-delineated grounding lines from the DInSAR data and split into $512\times 512$ pixel tiles (one pixel is $100\times 100$ m) to minimize GPU memory bottlenecks. We use overlapping staggered tiles to avoid artifacts at the boundaries. As a result, we have an augmentation factor of 8 (2$\times$ due to overlapping staggered tiles and 4$\times$ due to four directions) to help the neural network training. We end up with 5820 tile pairs for the training set on the Getz Ice Shelf and 4,507,501 around the Antarctic Ice Sheet. The outputs of the neural network have the same dimensions as the image tiles. To reconstruct larger scenes, we stitch the tiles back using a Gaussian averaging filter that places higher weight on the center of each tile, thereby avoiding edge effects.

### ML implementation

We use a fully-convolutional architecture for the semantic segmentation of grounding lines. Given the noisy and varied nature of the DInSAR data, we aim to directly segment the grounding lines, as opposed to classifying the various surfaces in each tile. The goal is to derive the probability of each pixel belonging to a grounding line or no grounding line. We use a 40-layer neural network with 966,119 trainable parameters, to perform the segmentation (see “Methods”). The larger size and complexity of this neural network attests to the noisier nature of the DInSAR data with features at multiple spatial scales compared to the simpler convolutional network previously used to delineate glacier calving fronts from optical imagery^15^. The neural network is trained on 5320 tiles. Each training epoch takes under 10 min on the Google Colab GPU accelerator, which is currently a Tesla P100-PCIe with 16 GB of memory. Using batch size of 30, the training is stopped after 9 epochs to avoid overfitting based on a minimum improvement threshold of 0.0001 in the value of the loss function applied to the randomly selected validation data in each epoch.

**Figure 2:**
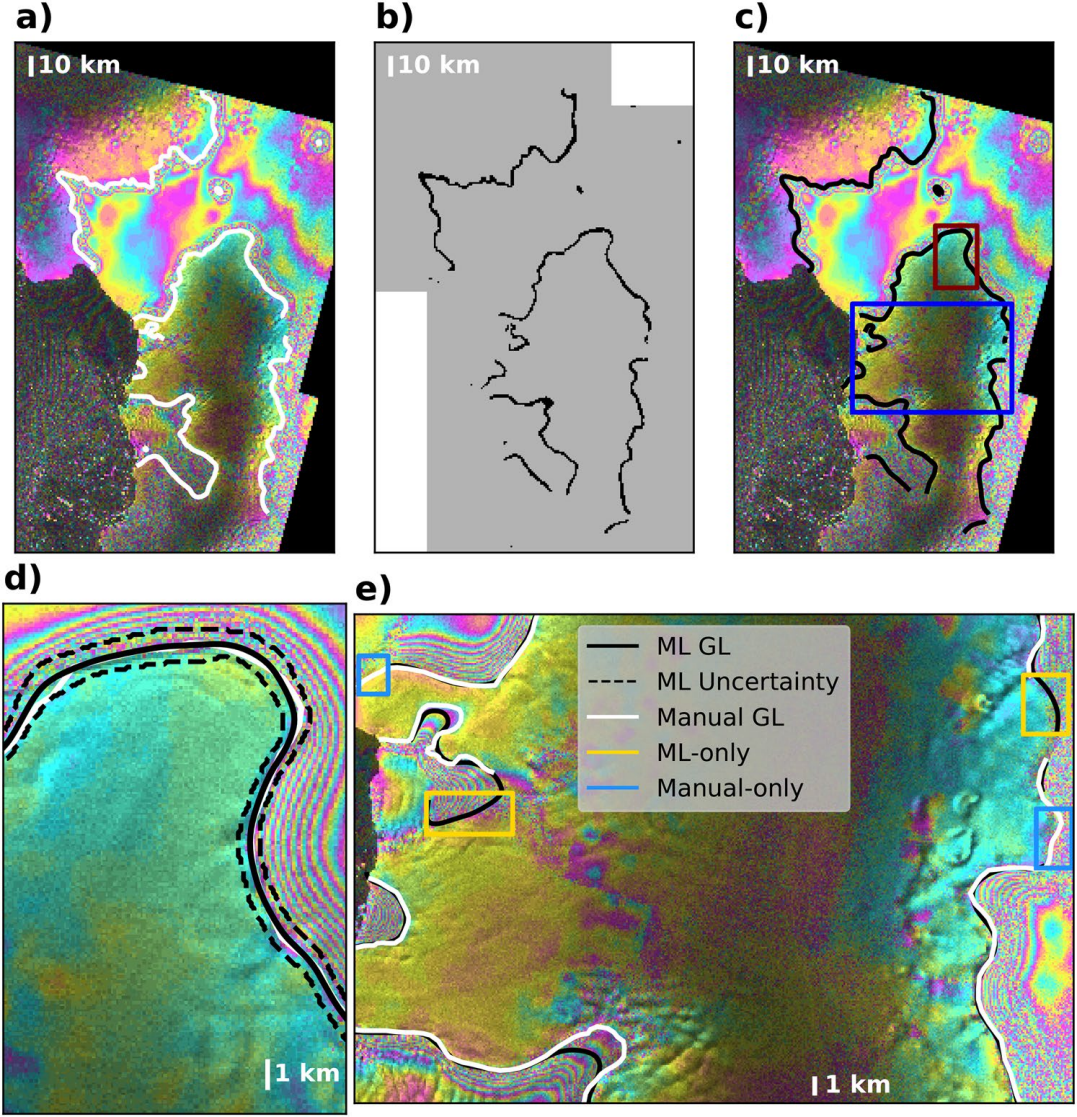
Different steps of the pipeline for (**a**) a sample interferogram with hand-drawn grounding line in white; (**b**) output of the neural network in raster format with a variable width that is a measure of uncertainty of the output. The gray mask in the background represents training (white) and testing (gray) sites; (**c**) Vectorized output and uncertainty contours; (**d**) zoomed-in region highlighted by the maroon box in panel (**c**) showing both manual and ML results and uncertainty bars; (**e**) Comparison of the manual and ML performances in the area outlined in blue in panel (**c**). The interferograms are obtained from Sentinel-1a/b data in 2018^13,14^, and are plotted with Python 3.7.4^17^ using Rasterio version 1.0.21^18^ and Matplotlib version 3.3.2^19^.

The fully-trained network is then tested on 500 new tiles. The neural network provides the probability of each pixel belonging to a grounding line. The original input data, the human-made training labels, and the output of the neural network are shown in Fig. 2. The gray mask indicates portions of the scene not used during training but used for evaluation. All delineated grounding lines belong to the test data set. The mask differentiates how the training and test data are selected randomly from the set of 5820 tiles.

To vectorize the results and extract grounding line positions, we set a threshold of 30% in the probability distribution around each grounding line to form a closed contour representing the uncertainty of the output (Fig. 2). By vectorizing the results, we introduce a deeper semantic understanding of the objects identified by the neural network. The contours are converted into polygons (See “Methods”), which may contain more polygons in the presence of pinning points, i.e. where the ice shelf is locally grounded due to a local rise in the sea floor^16^. We use the length of the perimeter of polygons to filter out noisy outputs. We use a threshold of 6 km to eliminate small features introduced by the network. The 6-km threshold is chosen to minimize the detection of spurious grounding lines, while avoiding the exclusion of real features. Residual errors remain, e.g. on Carlson and Evans glaciers feeding into the Ronne Ice Shelf where segments of both sides of the flexure zone are included in the grounding line detection. In other areas, e.g. Pine Island Glacier, we find short spurious line segments downstream of the grounding line. These errors, which comprise a small fraction of the total, are manually removed from the final product. The uncertainty estimates allow us to pinpoint the grounding line by drawing a centerline through the polygons (see “Methods”). These vectorized segments are the final output product, which we compare with the hand-drawn grounding lines (Fig. 2).

### Performance evaluation

In the analyzed areas, the ML-derived grounding lines are within the uncertainty bars of the hand-drawn grounding lines. To quantify the confidence in the performance of the ML pipeline, we use manual delineations not used during the training of the network. We calculate the mean difference between the neural-network and hand-drawn grounding lines by identifying an enclosing box around each delineated grounding line and finding the corresponding hand-drawn line. The distance to the opposite line is found by using the closest point on the opposite side. To avoid duplicates, we do not consider points that extend beyond the range of the reference line and map to the same end-point on the reference line. We find that the mean difference between the neural-network and hand-drawn grounding lines is 232 m, or 2.3 pixels, with a median absolute deviation (MAD) of 101 m, and an interquartile range (IQR) of 131 m.

In a few areas, the ML approach is inferior to the human interpretation, e.g. in the presence of pinning points^16^ which comprise narrow (in pixel size equivalent) and tide-dependent connections to the continent. In other areas, however, the ML interpretation is superior a posteriori to the human interpretation, for instance in areas of high noise where a conservative human interpreter would not identify a grounding line but would subsequently agree with the ML delineation (Fig. 2e). Human interpreters may apply various degrees of risk in drawing grounding lines, which creates inconsistencies in the data set, whereas the ML algorithm performs the identification objectively. We note that the manual delineation is often conservative. Other human experts may draw grounding lines in areas that are currently missing (e.g. blue boxes in Fig. 2e). We apply the same degree of caution to all manual delineations, including in the training data set. Yet, the ML algorithm correctly delineates GLs in these noisy areas despite the conservative training data.

Another advantage of the ML approach is to provide uncertainty bounds. Uncertainties are essential for modelers who need grounding line constraints in ice sheet models^20–22^. The uncertainty quantifies data noise. The uncertainty estimate is here provided by the width of the vectorized contours described previously. Considering the same test data from which the mean difference with manual delineations was derived, we find a mean uncertainty of 451 m, or 4.5 pixels, or twice the level of precision of the method. The manual delineation falls within the uncertainty bound of the ML results. The uncertainty varies with noise from 151 m in the best case to 1427 m in higher noise cases. Given a MAD of 110 m and an IQR of 155 m, most uncertainties are close to the minimum value.

### Antarctic grounding line delineation

**Figure 3:**
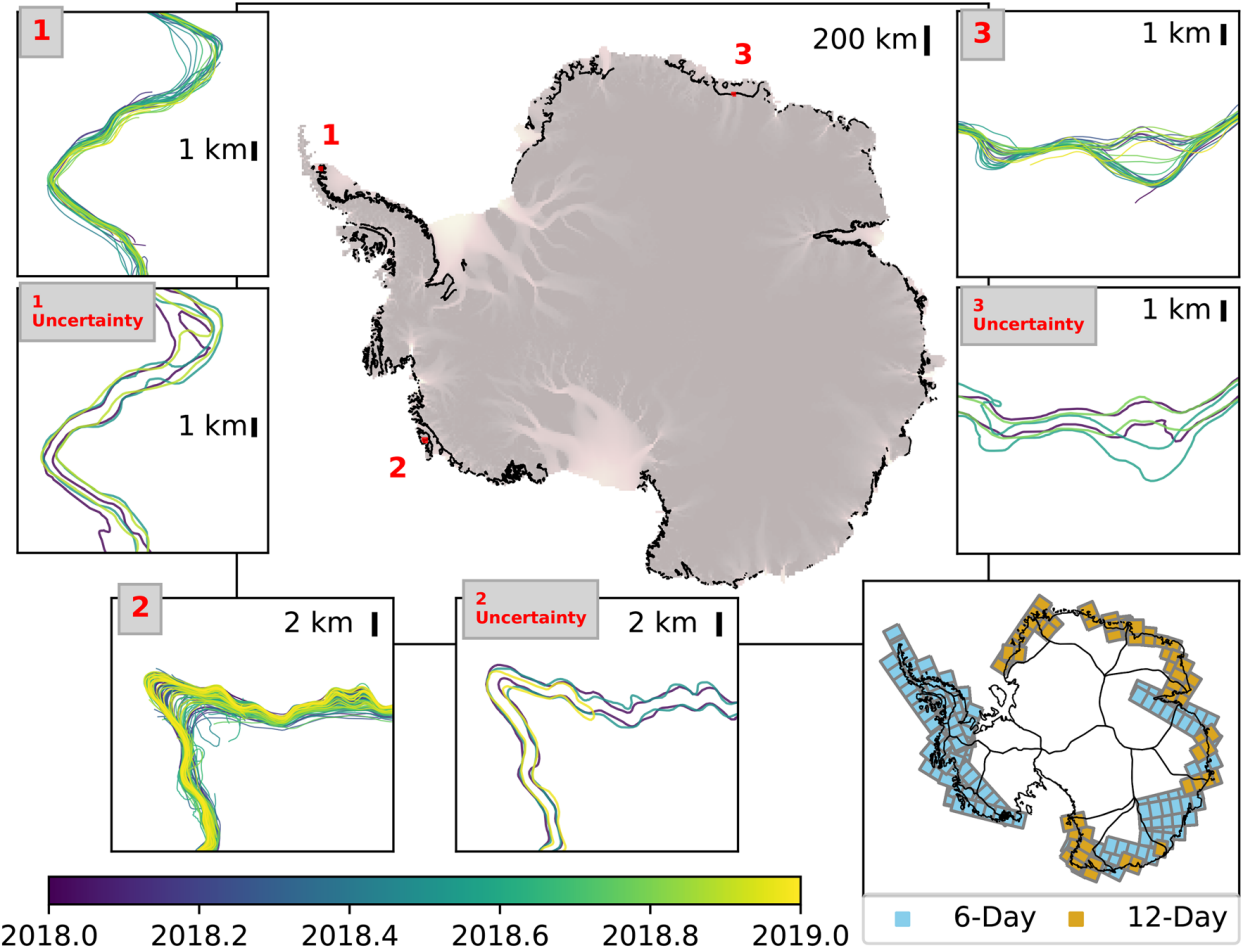
Complete grounding line delineation around the Antarctic Ice Sheet in the year 2018 superimposed on ice velocity; Location of all used Sentinel1-a/b tracks are shown in lower right with 6-day tracks in blue and 12-day tracks in dark orange. The insets show magnified GLs and three representative uncertainties separated by half a year for regions 1, 2 and 3 corresponding to the red boxes in the Peninsula, West Antarctica, and East Antarctica, respectively. The background ice velocity is obtained from the MEaSUREs data product^24–26^ and plotted with Rasterio version 1.0.21^18^ in Python 3.7.4^17^. The grounding lines and basin outlines in the insets are plotted with Matplotlib version 3.3.2^19^ in Python 3.7.4^17^. The basins are divided according to the Rignot drainage basin divisions^1,27–30^. The track information is plotted using Geopandas version 0.6.1^31^ in Python 3.7.4^17^.

We perform a delineation of 22,935 DInSAR interferograms around the Antarctic Ice Sheet (Fig. 3), which represents all 6-day and 12-day interval DInSAR interferograms acquired in year 2018 from Sentinel1-a/b. Running the 512 $\times$ 512 tiles through the pre-trained neural network takes about 17 ms per tile on average on the Google Colab GPU accelerator, accumulating to a total of $\sim$ 21 h for 4,507,501 tiles. Running the tiles on AMD 2.3 GHz CPUs takes on average 4 s per tile, accumulating to a total of 5000 CPU hours, which can be significantly sped up when run in parallel depending on the availability of the computational resources. For comparison, it would take a human interpreter up to 20 min to delineate a single interferogram, depending on the complexity of the scene, multiplied by 22,935 DinSAR interferograms, which adds up to 7600 h of continuous, labor-intensive, manual work. The complete grounding line and uncertainty data for the year 2018 is provided as Shapefiles for the 6-day and 12-day tracks at https://doi.org/10.7280/D1VD6G^23^.

## Discussion

### Performance

The performance of the ML algorithm is comparable to that of a human expert, but human interpretation is inherently more variable. For instance, human interpreters may place the grounding line closer to the last interferometric fringe or farther away depending on their own threshold of detection; or map a grounding line across an area of noise or refrain from doing it. Even an individual investigator will be hard-pressed to reproduce his/her own delineations repeatedly over time. The ML approach provides a more consistent and objective approach. To quantify the mean error introduced as a result of human delineation, we extracted the grounding line manually multiple times. We find a mean difference of 268 m. While the mean error is higher than that of the neural network, the spread is lower, with a MAD of 52 m and an IQR of 64 m. On the other hand, repeated delineations by the neural network will lead to consistent and repeatable results.

In many cases, the network performs an a-posteriori judicious mapping in places where a conservative human interpreter may be more cautious and yet subsequently agree with the ML result, as shown in Fig. 2e. There are comparatively fewer cases where the neural network misses portions of the grounding line delineated by human interpreters, except where the grounded area is only a few image pixels in size (Fig. 2), in which case the neural network may omit part of the boundaries or miss the ice rise entirely during post-processing, noise-removal procedures. The limitations of the ML approach in the presence of small branches and islands could be alleviated by processing the data at a finer sample spacing. Overall, the ML technique identifies more grounding line segments than the human interpreter can because the loss function of the neural network penalizes false negatives (no grounding line) more severely than than false positives (grounding line) and because human interpreters are often conservative.

### ML behavior

**Figure 4:**
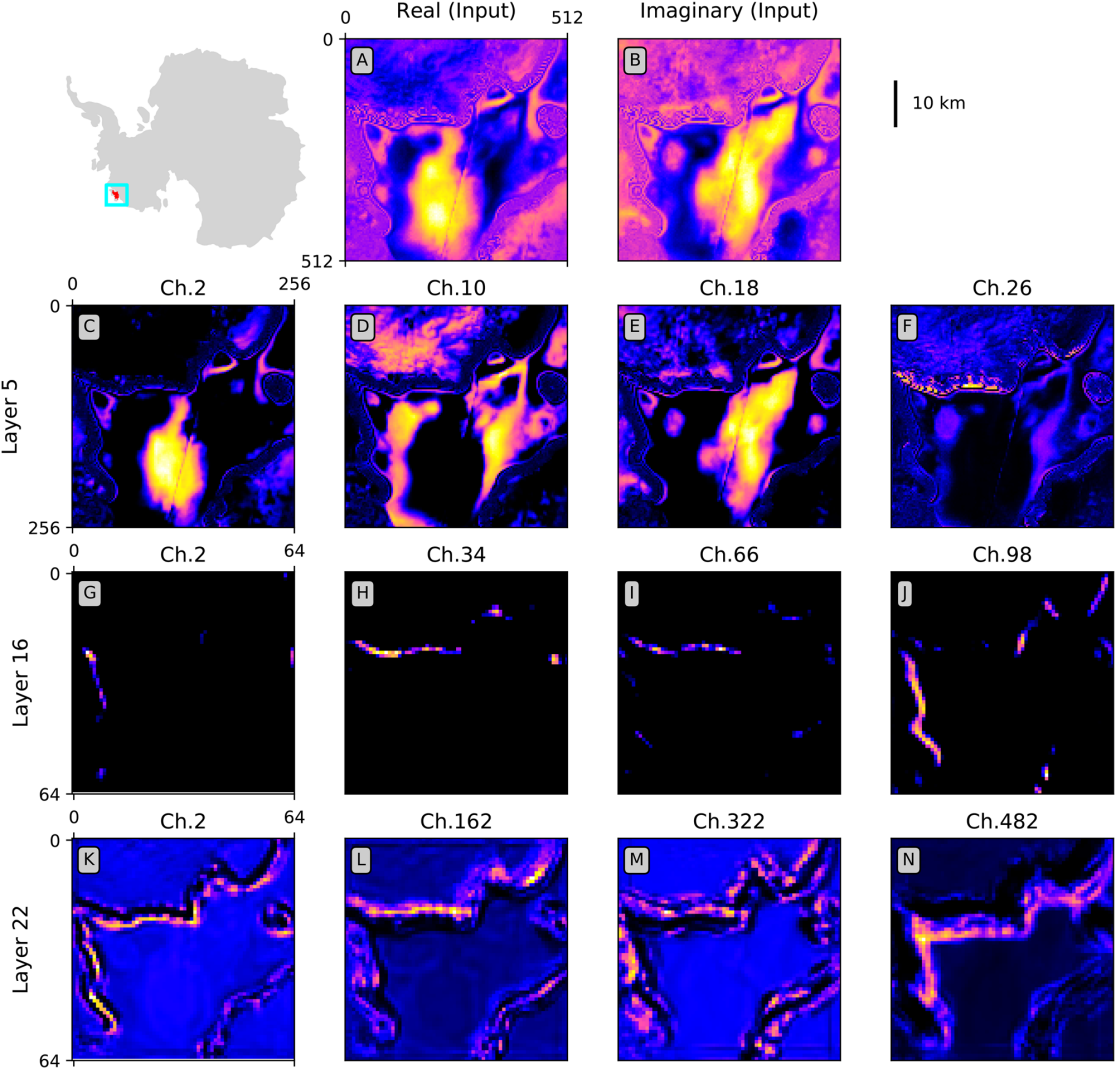
Activation Maps for the 1st (input), 5th, 16th, and 22nd layers. Note that magnitude of values in the intermediary layers before the final sigmoid layer depend on the choice of the activation function and do not have physical meaning. The numbers of channels represented are indicated on top of each plot. Note the decreasing image size as evident by the number of pixels shown on the axes. The location of the image is show on the upper left corner for text. The scalebar for the input is given on the upper right corner. The dilation rates for Layer 22 are 1, 2, 3, 4 for channels 22, 164, 322, and 482, respectively. The activation maps and the labels on the inset of Antarctica are plotted using Matplotlib version 3.3.2^19^. The Antarctic map is created using the default low resolution Earth map in Geopandas version 0.6.1^31^ and plotted in Antarctic polar stereographic (EPSG:3031) projection. All libraries are used with Python 3.7.4^17^.

To better understand the behavior of the neural network, we examine the activation maps produced by the layers of the network (Fig. 4). These layers refer to the output produced once convolutional kernels are applied to a given layer. By studying what features are picked up by the network, we gain a better understanding of the information used by the algorithm for the delineation of grounding lines.

The first row shows the two input channels (real and imaginary components of the interferogram). The activation maps in the second row correspond to the fifth layer of the network (Panels C–F). For each row, we divide the channels into quarters and showcase four activation maps at equally spaced intervals. At layer 5, the network picks up large scale features that are not necessarily relevant to the grounding line, and artifacts, e.g. faint diagonal feature across the interferogram on the floating ice tongue. This highlights the need for a deep network capable of encapsulating the many features of the data, hence the relatively large depth of the neural network. Panels G–J show the activation maps of layer 16, which is the convolutional layer with dimensions $64\times 64\times 128$ before the parallel atrous layers. While the grounding line is starting to emerge at this stage and the noise is diminished, the network is not able to detect both the large-scale features and the fine details necessary for detecting grounding lines. In addition, each activation map at this stage only exhibits certain aspects of the grounding line, but not the entire line in all spacial orientations. The multi-scale problem is resolved by the use of a series of parallel atrous convolutions with dilation rates of 1 to 5 (layers 17–21 in Table S2). Each of the dilated kernels picks up desired features at different spatial scales without additional computational costs. To further reduce the number of parameters, we use depthwise-separable convolutions^32^. The activation maps for the concatenated results of the atrous convolutions, given by layer 22 with dimensions $64\times 64\times 640$, show grounding lines without the additional undesired features from the previous layers (Panels K–N). This layer is the densest representation of the features, from which the full-scale image is reconsidered through a series of upsampling and convolutional layers.

The network distinguishes two sides of the grounding zone, represented by asymmetric bands in the activation maps at the boundaries of the grounding zone (Fig. 4). The network picks up the correct side of the grounding zone as the grounding line, without the need for information on flow direction which could be problematic in areas of very slow motion. Panels from K to N correspond to increasingly dilated convolutional kernels. Panel K, with a dilation rate of 1, shows high spatial details with relatively more noise. Panel N, with a dilation rate of 4, picks up the large-scale features with a large field of view. While Panel N does not contain as much spatial detail, it retrieves the most generic features with less noise. By combining these multi-scale views and putting them through additional convolutional and upsampling layers, we generate an image of grounding lines only.

### Grounding zone width

**Figure 5:**
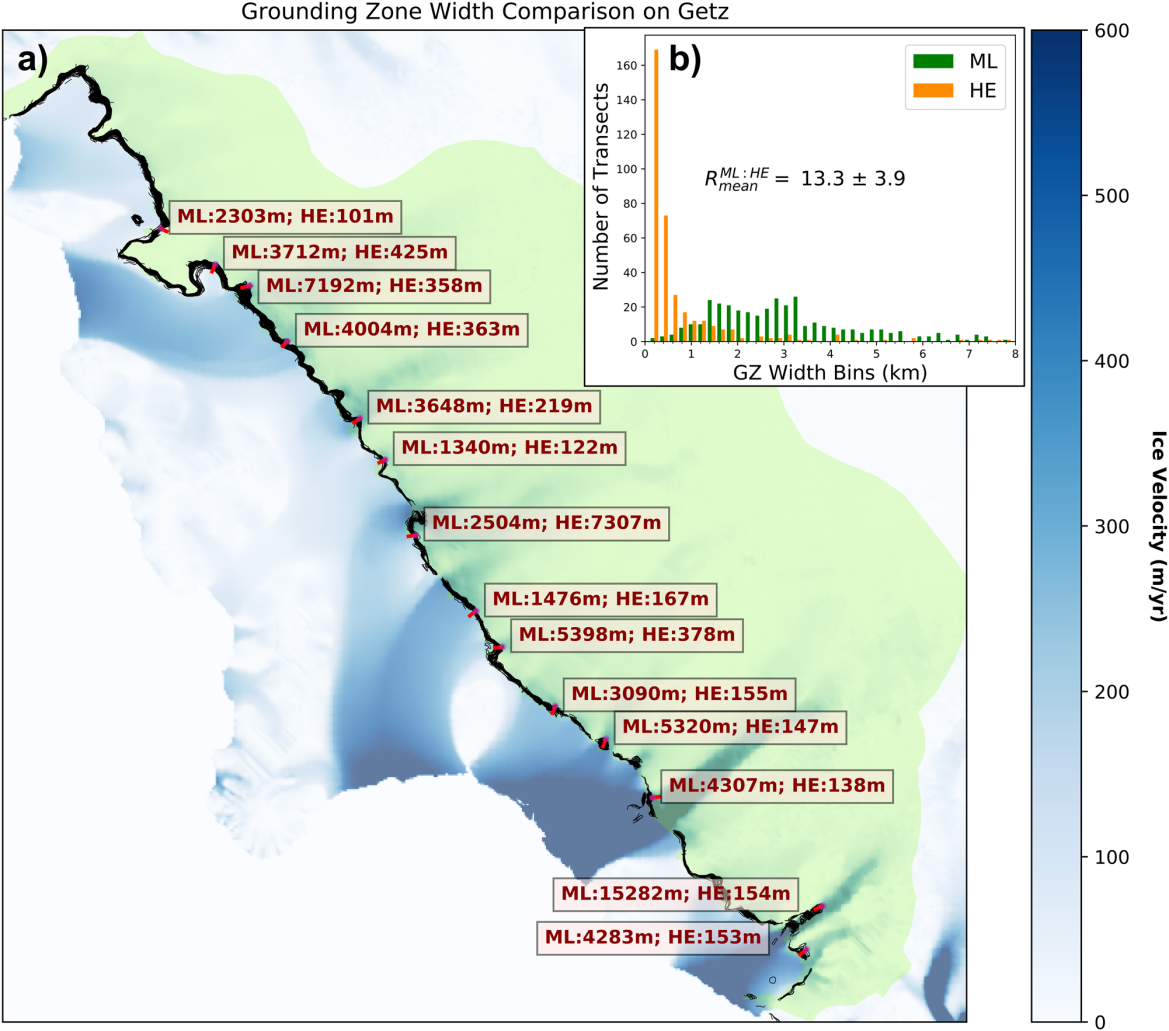
Comparison of the predicted width of the grounding zone from hydrostatic equilibrium (HE) versus the observed width from machine learning (ML) along Getz Ice Shelf, West Antarctica. (**a**) Sample 14 comparison sites along the coast. Getz Basin is green shading. Ice speed^24^ is color-coded in blue. The 2018 grounding lines are black. The delineated (*ML*) and expected (*HE*) widths are quoted in red letters along the flow direction (red line). (**b**) HE vs. ML GZ width less than 8 km for 500 transects along the coast. R$^{ML:HE}_{mean}$ is the mean ratio of ML to HE width. The ice speed field is obtained from the MEaSUREs data product^24–26^ and plotted with Rasterio version 1.0.21^18^. The grounding lines, width labels, and the histogram in the inset are plotted with Matplotlib version 3.3.2^19^. The Getz basin is drawn according to the Rignot drainage basin divisions^1,27–30^ and is plotted using Descartes version 1.1.0^37^ and Matplotlib version 3.3.2^19^. All libraries are used with Python 3.7.4^17^.

By analyzing delineations across the entire ice sheet, we find that the grounding zone, which is the area over which a grounding line moves back and forth with changes in oceanic tides, is several km in width. In the case of interaction of solid ice on a hard bed, the level of migration of the grounding line with tide is dictated by hydrostatic equilibrium, which depends on both surface and bed slopes^33,34^. With slopes of the order of 1% and tides in the range of 1 m, we expect migrations of the order of 100 m. Instead, we detect migrations of several kilometers, well beyond the uncertainty in slopes. To quantify this discrepancy, we calculate the grounding zone width based on hydrostatic equilibrium as in Tsai and Gudmundsson^35^ along the Getz Ice Shelf, in West Antarctica, where we have continuous and high-quality delineations with dense coverage of 6-day interferograms (Fig. 3). The expected grounding zone width, referred to as the *HE* (Hydrostatic Equilibrium) grounding zone, is calculated as

1 $$\begin{aligned} \Delta L_{HE} = \Delta h \left[ \beta + \frac{\rho _i}{\rho }(\alpha -\beta ) \right] ^{-1} \end{aligned}$$

where $\Delta h$ is the tide height, $\beta$ is the bed slope, $\alpha$ is the surface slope, and $\rho _i$ and $\rho$ are the density of ice and seawater, respectively^35^. Note that the downstream migration distance is 9 times smaller than the upstream distance. Here, we use the upper case as a conservative estimate of the grounding zone width. In addition, we assume a uniform 2 m tide height to get an upper bound of the *HE* grounding zone. We calculate $\beta$ and $\alpha$ along the direction of flow indicated by ice velocity vectors from MEaSUREs^24–26^ using the surface and bed topography from BedMachine v1^36^, over a spatial scale of 3 km, which is several times the mean ice thickness. We compare the result with the observed width of the grounding zone measured along the same ice flow direction. A set of comparison points are shown in Fig. 5.

We find that the observed grounding zone width is one order of magnitude larger than the width expected from HE. Berry Glacier is an exception with a grounding zone width two orders of magnitudes wider than expected. The inset (Fig. 5b) shows the distribution of expected widths, which indicates a consistent pattern. The results are skewed towards larger widths. The width from HE is typically less than 1 km. The delineated (ML) is on average 13.3 ± 3.9 larger than the width from HE, or one order magnitude.

Broader grounding zones are predicted when ice interacts with a deformable bed^38^ instead of a hard bed, or if un-grounding of ice is interpreted as the propagation of an elastic crack at the grounding line^35^. Our broader mapping exercise indicates that Getz Ice Shelf is not an isolated case but the norm in Antarctica, i.e. grounding zones are typically several km wide and are not well represented from hydrostatic equilibrium on a hard bed^39–41^. This result has implications for ice sheet models who treat grounding lines with hydrostatic equilibrium.

### Implications

A consequence of wider grounding zones is that seawater must intrude over considerably longer distances beneath grounded ice at high tide than expected. Water intrusions in turn imply that ice probably melts at tidal frequencies in these broad regions. This information is relevant to ice sheet models because wider grounding zones and the possibility of high ice melt rates within the grounding zone will make the model more sensitive to ocean thermal forcing and the glaciers retreat faster in warming scenarios^42^. Conversely, in areas of steep slopes or abrupt transition boundary where the grounding zone is narrow and seawater intrusion is likely to be limited, a treatment with hydrostatic equilibrium and no melt at the grounding line is probably satisfactory.

The application of the ML method to the entire Antarctic Ice Sheet provides a unique and novel data set for the scientific community. The results confirm that wide grounding zones are the norm. Due to the uneven and sparse temporal sampling of the data, we cannot analyze changes within a tidal cycle, or from month to month, and only examine aggregated changes over an entire year at present. The ML approach detects the grounding line with a precision of 200 m and the width of the grounding zone over a tidal cycle. This precision is sufficient for large scale ice sheet numerical models. We also find that the approach is scalable to pan-Antarctica without retraining the network, at high speeds of computation compared to manual interventions. Visual inspection of the results in other areas, e.g. Pope, Kohler, and Smith (Fig. 3), confirms that the ML approach is reliable over the remainder of the ice sheet periphery.

The complete mapping of grounding lines around Antarctica performed by the ML algorithm provides a new basis for the analysis of grounding line dynamics in Antarctica that was not available previously. We expect that the product from year 2018 to be of considerable interest for the scientific community, in particular numerical ice sheet modelers and ocean modelers, and will help improve projections of ice sheet evolution and contributions to sea level rise from models. We anticipate to perform this delineation routinely with Sentinel-1a/b and other InSAR data, e.g. the Italian CSK-2G, the Japanese ALOS PALSAR-2, -3 and -4 and the upcoming NASA/Indian Space Research Organization (ISRO) NISAR mission.

## Methods

Images are split into overlapping tiles that avoid edge effects. Partially overlapping tiles in four directions combined with a staggered grid produces 8 partial coverings of every area as a form of augmentation. We generate 5320 tile pairs for training. We use a 40-layer deep encoder-decoder convolutional neural network with parallel atrous convolutional layers (Atrous Spatial Pyramid Pooling^43^), inspired by the DeepLabv3+ architecture^44^. In order to minimize the number of trainable parameters, we use depthwise-separable convolutions^32^ for the parallel atrous layers, but we find that extending depthwise-separable convolutions to all layers degrades the performance of the network. We add skip connections between encoding and decoding layers to convey contextual information, as in^45^. All convolutional layers use the Expontential Linear Unit (ELU) activation function^46^, with the exception of the last convolutional layer with a sigmoid activation function producing the predicted probability of the class of each pixel. In total, we have 966,119 trainable parameters in the models. Descriptions of all layers are provided in Table S2. Given the large class-imbalance between non-GL to GL pixels, we use a weighted binary cross-entropy loss function to penalize false negatives more harshly than false positives ($L=-\frac{1}{N}\sum _i^N[Ry_i\log (\hat{y}_i)+(1-y_i)\log (1-\hat{y}_i)]$, where *N* is the number of pixels, *R* is the penalization ratio which is 727 based on the average ratio of GL vs non-GL pixels, $\hat{y}_i$ is the prediction value for pixel *i* between 0 and 1, and $y_i$ is the binary GL/non-GL “truth” label for pixel *i* from the human-made training dataset). The neural network provides raster images of the GLs, which are vectorized by drawing closed contours around GL pixels using a threshold of 0.3 (30% probability of pixel belonging to GL class). The width of these contours provide the uncertainty estimate for the neural network output. In order to draw the centerline through the contours, we use the label_centerlines Python package.

## Supplementary Information

Supplementary Information.

## Data Availability

The dataset generated during and analysed during the current study are available in the UC Irvine Dryad repository, https://doi.org/10.7280/D1VD6G^23^. The algorithm described in the manuscript, model configurations, and the trained model are accessible on the associated Github repository found at https://github.com/yaramohajerani/GL_learning.
