# Supplementary material for: Automatic delineation of glacier grounding lines in differential interferometric synthetic-aperture radar data using deep learning
Source: Sci Rep. 2021 Mar 2;11:4992. doi: 10.1038/s41598-021-84309-3 (PMC7925556; doi:10.1038/s41598-021-84309-3)
Supplement: Supplementary file 1 — Supplementary Information. [file 41598_2021_84309_MOESM1_ESM.pdf]

Supplementary Material: Automatic Delineation of Glacier Grounding Lines in Differential Interferometric Synthetic-Aperture Radar Data using Deep Learning

Yara Mohajerani[1,2,*], Seongsu Jeong[1], Bernd Scheuchl[1], Isabella Velicogna[1,3], Eric Rignot[1,3], and Pietro Milillo[1]

[1]University of California Irvine, Department of Earth System Science, Irvine, CA, 92697, USA
[2]University of Washington, eScience Institute and Department of Civil and Environmental Engineering, Seattle, WA, 98195, USA
[3]Jet Propulsion Laboratory, Pasadena, CA, 91109, USA
[*]ymohajer@uci.edu

S1 Data: Sentinel1-a/b 2018 Tracks and Tiles

All Sentinel-1a/b DInSAR 6-day and 12-day tracks from 2018 were utilized in the study. The breakdown of the track numbers, number of interferograms, and the number of processed 512×512 tiles is shown in Table S1.

S2 Neural Network Architecture

A 40-layer deep convolutional neural network with 966,119 tunable parameters is used to delineate the grounding lines, as described in the Methods section of the main text. All the layers, corresponding dimensions, and inputs are outlined in Table S2.

S3 Antarctic Grounding Line Data

The dataset produced as a part of this study containing all the Antarctic grounding lines for the year 2018 are deposited into the UC Irvine Dryad data repository as Shapefiles. Four sets of files are provided, corresponding the the 6-day and 12-day tracks and the corresponding grounding line uncertainties as described in the main text. The data can is accessible at `https://doi.org/10.7280/D1VD6G`[4].

References

1. Chen, L.-C., Papandreou, G., Schroff, F. & Adam, H. Rethinking atrous convolution for semantic image segmentation. *arXiv preprint arXiv:1706.05587* (2017).
2. Chen, L.-C., Zhu, Y., Papandreou, G., Schroff, F. & Adam, H. Encoder-decoder with atrous separable convolution for semantic image segmentation. In *Proceedings of the European conference on computer vision (ECCV)*, 801–818 (2018).
3. Chollet, F. Xception: Deep learning with depthwise separable convolutions. In *Proceedings of the IEEE conference on computer vision and pattern recognition*, 1251–1258 (2017).
4. Mohajerani, Y., Jeong, S., Scheuchl, B., Velicogna, R. E., I. & Milillo, P. Data from: Automatic delineation of glacier grounding lines in differential interferometric synthetic-aperture radar data using deep learning. *UC Irvine, Dataset*, https://doi.org/10.7280/D1VD6G (2021).

6-day Tracks	# of 6-d interferograms	# of 6-d tiles	12-day Tracks	# of 12-d interferograms	# of 12-d tiles
3	1,519	360,844	2	163	40,308
7	1,492	215,852	10	77	17,872
10	1,152	251,809	14	96	17,460
11	741	87,297	15	60	8,144
37	818	109,577	28	342	83,720
38	1,663	378,116	41	228	40,248
40	524	73,689	43	228	50,272
50	144	29,401	46	177	42,380
52	745	134,001	50	365	84,620
53	921	221,113	54	105	23,152
54	315	72,417	58	50	7,488
55	484	69,633	59	140	19,612
65	1,566	300,484	69	173	28,260
68	280	72,073	70	114	18,144
69	1,220	206,221	72	399	87,184
70	997	173,129	74	107	22,208
82	942	220,017	87	100	19,464
85	528	118,020	100	114	27,136
98	563	98,480	102	86	9,136
99	417	71,936	129	114	25,456
134	710	168,092	136	27	5,536
169	1,769	375,752	146	46	5,796
			164	114	15,952
**6-d Total**	19,510	3,807,953	**12-d Total**	3,425	699,548
**All Tracks Total**	22,935	4,507,501			

Table S1. Breakdown of the DInSAR data utilized in the analysis of 2018 grounding lines across the Antarctic ice sheet. Data is separated into 6-day and 12-day interferograms. Then tile columns refer to the number of 512×512 tiles processed by the neural network, which are augmented by having multi-directional and overlapping patches, as described in the main text.

Layer #	Type	Output Shape	Input Layer(s)
1	Input	512×512×2	
2	Convolution (3×3, ELU)	512×512×32	1
3	Dropout (0.2)	512×512×32	2
4	Convolution (3×3, ELU)	512×512×32	3
5	Max Pooling	256×256×32	4
6	Convolution (3×3, ELU)	256×256×64	5
7	Dropout (0.2)	256×256×64	6
8	Convolution (3×3, ELU)	256×256×64	7
9	Max Pooling	128×128×64	8
10	Convolution (3×3, ELU)	128×128×64	9
11	Dropout (0.2)	128×128×64	10
12	Convolution (3×3, ELU)	128×128×64	11
13	Max Pooling	64×64×64	12
14	Convolution (3×3, ELU)	64×64×128	13
15	Dropout (0.2)	64×64×128	14
16	Convolution (3×3, ELU)	64×64×128	15
17	Depthwise-Separate Conv (3×3, ELU) - Dilated ×1	64×64×128	16
18	Depthwise-Separate Conv (3×3, ELU) - Dilated ×2	64×64×128	16
19	Depthwise-Separate Conv (3×3, ELU) - Dilated ×3	64×64×128	16
20	Depthwise-Separate Conv (3×3, ELU) - Dilated ×4	64×64×128	16
21	Depthwise-Separate Conv (3×3, ELU) - Dilated ×5	64×64×128	16
22	Concatenate	64×64×640	17-21
23	Upsampling	128×128×640	22
24	Concatenate	128×128×704	23,12
25	Convolution (3×3, ELU)	128×128×64	24
26	Dropout (0.2)	128×128×64	25
27	Convolution (3×3, ELU)	128×128×64	26
28	Upsampling	256×256×64	27
29	Concatenate	256×256×128	28,8
30	Convolution (3×3, ELU)	256×256×32	29
31	Dropout (0.2)	256×256×32	30
32	Convolution (3×3, ELU)	256×256×32	31
33	Upsampling	512×512×32	32
34	Concatenate	512×512×64	33,4
35	Convolution (3×3, ELU)	512×512×32	34
36	Dropout (0.2)	512×512×32	35
37	Convolution (3×3, ELU)	512×512×32	36
38	Convolution (3×3, ELU)	512×512×3	37
39	Convolution (1×1, Sigmoid)	512×512×1	38
40	Flatten	262144×1	39

Table S2. Full outline of the layers of the neural network. All convolutional layers have a ELU (Exponential Linear Unit) activation function with 3×3 convolutional kernels, with the exception of layer 39, which uses a sigmoid activation function and 1×1 convolutional kernels. All dropout layers use a dropout fraction of 0.2. Layers 17 to 21 are parallel layers all connected to layers 16 and 22, as part of the Atrous Spatial Pyramid Pooling[1,2]. In addition, these 5 parallel convolutional layers use depthwise-separable convolutions[3] to reduce the number of training parameters, as well as dilation rates of 1 to 5, respectively, for the dilated/atrous convolutions. Layer 40 of the network simple reshapes the output to a flattened array without any transformation to the values.
